# The Malaria Atlas Project: Developing Global Maps of Malaria Risk

**DOI:** 10.1371/journal.pmed.0030473

**Published:** 2006-12-05

**Authors:** Simon I Hay, Robert W Snow

## Abstract

The primary goal of the recently launched Malaria Atlas Project is to develop the science of malaria cartography.

In allocating public health resources, the guiding principle should be an evidence-based quantification of need. A significant effort to categorize diseases by their global morbidity and mortality impact has developed during the last decade, epitomized by the Global Burden of Diseases [1] and the Disease Control Priorities projects [2]. But despite these efforts, the evidence base for allocating resources for malaria control on a global scale is poor.

National reporting on malaria continues to be fanciful; Kenya, for example, reported only 135 malaria deaths in 2002 to the World Health Organization [3]. In addition, less than half (22/49) of the malaria-endemic countries in Africa provided information for the most-recent reporting year, 2003; the rest were older [3]. Information on the global burden of malaria remains the subject of best guesses rooted in national reporting systems [3], informed estimation based on epidemiological data linked to historical malaria distributions [4], or unvalidated models of malaria distribution in Africa [5–7]. As a corollary, resource allocations for malaria interventions remain driven by perceptions and politics, rather than an objective assessment of need. This status quo is untenable when global and national financial resources must be defined to meet needs for new, expensive antimalarial drugs and commodities to prevent infection, and to ensure that these interventions are optimally targeted.

It has been almost 40 years since the last global map of malaria endemicity was constructed [8], and a decade since the need for maps of malaria transmission in Africa was first advocated [9]. Although substantial progress has been made [10–21], an evidence-based map of malaria transmission intensity for Africa remains illusive, and there have been no recent efforts to construct a credible evidence-based global malaria map.

**Figure:**
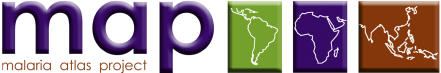
MAP Logo

## A New Mapping Project

The primary goal of the recently launched Malaria Atlas Project (MAP) is to develop the science of malaria cartography. Our approach will be first to define the global limits of contemporary malaria transmission; we have initiated this process [12,13], but will substantially refine these layers with additional medical intelligence in future years.

Within these limits, we plan to then model endemicity using a global evidence base of malaria parasite prevalence. This Health in Action concentrates mostly on how we intend to achieve this important goal. Once we have created these global endemicity maps, these will then provide a baseline to facilitate estimation of populations at risk of malaria and more-credible predictions of disease burden. These maps will also provide a platform to help target intervention needs, and may provide a means to measure progress toward national and international malaria public health goals at a global scale.

## Why Do We Need Maps of Malaria Transmission?

Malaria parasite transmission intensity is spatially heterogeneous [6,22–24]. This heterogeneity has important implications for risks and age patterns of progression from malaria infection to disease, disability, and death [5,25].

Endemicity is a measure of the level of malaria challenge in a human population, and determines the average age of first exposure, the rate of development of immunity, and thus, the expected clinical spectrum of disease [25,26]. Therefore, suites of relevant interventions should be tailored to these basic epidemiological foundations [9,27–29].

This is obvious for malaria early warning systems, for example, that have a rationale only in epidemic-prone areas [30,31]. In addition, intermittent presumptive treatment of infants is likely to have little impact on the incidence of clinical malaria and anaemia in areas of exceptionally low transmission [32]. Moreover, where one should withhold iron supplementation in young children demands an understanding of the balanced risks of iron deficiency, malaria disease incidence, and intensity of transmission [33,34]. Furthermore, optimizing the introduction of diagnostics to rationalize the use of new, expensive therapies will require better tools to target where this is cost efficient and where presumptive treatment remains appropriate. We anticipate that other interventions are likely to have health impacts and cost-effectiveness balances that may vary under different endemicity conditions, and we propose to conduct a detailed systematic review of the evidence.

It is often not immediately apparent when reading guidelines for malaria control that there are many intervention options available, that these may need to be appropriately combined, and that the optimal mix could depend on the intensity of malaria transmission in a given area. This would be as true for Africa as it is for other malarious territories of the Old and New Worlds. Global maps of malaria endemicity should therefore be essential in every step, from selecting appropriate intervention options and identifying requirements and budgeting, to planning, implementing, and monitoring at subnational, national, and regional scales.

**Box 1. The MAP Web Site**

The MAP Web site (http://www.map.ox.ac.uk) was launched on May 1, 2006, to further the aims and ambitions of MAP. The Web site allows users to visualize the current distribution of the assembled PR data through static maps in Web browsers, or more interactively through “.kmz” files that enable the data to be displayed in Google Earth (http://earth.google.com). We are currently interested in gathering additional PR data from the public health community, and to facilitate communication we have translated the entire Web site into Spanish and French.

MAP is different than previous attempts at mapping malaria, primarily because it is a global initiative, but also because it aims to share data from the outset. Those supplying useful PR data will be provided with the full database for their country of interest, provided full permission is granted from the data owners for distribution. In addition, the entire database will be released in the public domain after component outputs have been peer reviewed. We have set a June 1, 2009, deadline for this release.

A second unique feature of MAP is that it operates with strict inclusion criteria for PR data: only random or complete community-sample surveys conducted post-1985, where parasite species and age groups are defined and the survey involves more than 50 persons to minimize sampling error [68]. Extensive details of these and additional inclusion rules are provided online in English, Spanish, French, Chinese, and Swahili.

Thirdly, the MAP project will collect data on P. falciparum malaria, as well as the often neglected P. vivax parasite. The Web site also allows formal acknowledgment of those interested individuals and institutions who contribute data. We encourage you to have a look and send us feedback at map@zoo.ox.ac.uk.

## Large Area Efforts to Map Malaria since the 1960s

The fuzzy climate-suitability map for stable Plasmodium falciparum malaria transmission was a milestone in the mapping of malaria in Africa [10]. It represented the first attempt for several decades to provide a map of P. falciparum transmission at a continental scale, and has been widely used and cited by scientists, international agencies, and national malaria control programmes [6,35,36].

However, it has also been widely misinterpreted, as it represents a measure of the likelihood that stable transmission can occur, rather than ranges of transmission intensity. Furthermore, it has never been formally evaluated against contemporary parasite rate (PR) data outside of Kenya [37]. What is required for defining both disease risks and intervention need is a spatial model that predicts levels of endemicity, defined and validated by empirical data and constructed at a global scale. This approach to assembling epidemiological “training” data, environmental “predictor” data, and a suite of statistical mapping techniques to relate the two is considered below.

## An Archive of Parasite Prevalence

There are many ways to measure the abundance of malaria in a given location, and they all have their advantages and disadvantages that have been reviewed elsewhere [23,24,38]. Regardless of any epidemiological preferences, PR data indisputably constitute the bulk of the global information available on the distribution of malaria endemicity. The PR is the proportion of a sampled population that is confirmed positive for malaria parasites, canonically by identifying immature “ring stage” trophozoites in blood slides [39].

We have adopted a single and traditional classification of malaria endemicity based on the PR [40], to standardize our definition of risk globally. Endemicity is defined by the PR in the two- to ten-year age cohort (hypoendemic, less than 0.1; mesoendemic, 0.11–0.5; hyperendemic, 0.51–0.75), except for the holoendemic class (greater than 0.75) where the PR refers to the one-year age group [40]. This is important because “risk” is a geographically relative concept: nation states in Latin America identify areas of “high” risk that would be classified as low risk in sub-Saharan Africa.

**Figure 1:**
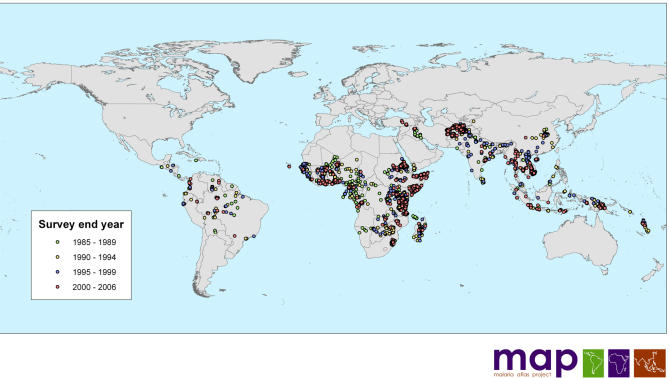
Distribution of the *n* = 3,036 PR Data Points Collected and Geopositioned by September 10, 2006 There are *n* = 420 PR surveys conducted in the 1985–1989 period, *n* = 557 in the 1990–1994 period, *n* = 556 in the 1995–1999 period, and *n* = 1,503 in the 2000–2006 period.

To gather global data on PR surveys of a sufficient extent and density to generate endemicity surfaces at moderate spatial resolution requires combinations of traditional and nontraditional search strategies. This process has involved electronic searches of formal literature and grey literature databases, as well as using personal contacts with malaria research scientists and malaria control personnel. Most recently, we have developed a Web site to guide people in identifying additional data sources from areas where information is lacking (Box 1). As of September 10, 2006, our search has provided 3,036 spatially independent geopositioned PR surveys undertaken since January 1985 from an aggregate sample of 2,143,979 blood slides in 79 malaria-endemic countries. The data included 2,728 survey locations reporting P. falciparum prevalence (Figure 1) and 1,379 locations reporting P. vivax prevalence.

In addition, the distribution of the main Anopheles malaria vectors and the frequency of the inherited haemoglobin disorders will constrain malaria infection risks and disease outcomes globally. Both will be subject to a similar intensity of data search and assembly, which will be described elsewhere.

## An Archive of Global Environmental Data

Malaria is a vector-borne disease and the culpable anopheline mosquitoes are very sensitive to climate. This has been exploited in many ways to predict the distribution of malaria in time [14,30,31,41,42] and space [15,20,43]. Mapping the distribution of malaria requires spatially referenced data (e.g., altitude, temperature, rainfall, and vegetation extent) to be matched to the PR-survey positions, to establish uni and/or multivariate statistical relationships between malaria endemicity and the environment. These relationships can then be applied to the environmental data, in more or less sophisticated ways (see below), to generate continuous global maps [20].

Our proclivities are for such information derived from Earth-observing satellites [44–48] because they are globally consistent measurements, often more contemporary and often of higher spatial resolution than interpolated climatologies [47]. Examples of these environmental data [47] have been made available in the public domain, and will be hosted on a MAP Web site when all necessary distribution rights have been negotiated. There is significant potential for improvement in these environmental themes through data collected by new generations of satellite sensors [48]. Continual improvement of these data will be part of the ongoing commitment of MAP.

## First Steps to Maps of Force of Infection

As a cross-sectional measure of prevalence, the PR is a less direct measure of malaria transmission than the entomological inoculation rate (the number of infective bites per capita, often expressed annually for P. falciparum, hence APfEIR) [22,23], the vectorial capacity (canonically, *C*) or the basic reproductive number (canonically, *R* *_0_*) [38]. These “force of infection” metrics, however, are much less frequently measured, [6,22] and while not recorded at sufficient frequency to enable mapping, will be archived by the MAP as companion data to inform modelling.

Moreover, in high-endemicity areas, PR samples are often restricted to children, but in areas of low endemicity, surveys are usually extended to include all age groups. PR is therefore confounded by the interacting factors of the age of the population sampled, its immune status, and the “detectability” of peripheral parasitaemia [23,24]. It is necessary to transform PR into a measure of the force of infection of malaria controlling for these factors. This is because these interrelated measures are more closely related to the life-history characteristics and dynamics of the Anopheles vector populations that we will attempt to model with environmental data. Our goal is to generate APfEIR, *C*, and ultimately *R* *_0_* surfaces from our PR data for mapping, and the modelling framework within which to perform these conversions has already been developed [23,24].

Those techniques required to standardize PR for age represent an ongoing challenge, although methods by which to achieve this have been suggested [11]. The models written to perform these conversions will be made freely available in the public domain pending peer review. R is the chosen MAP platform as it is a programming environment that is free to all (http://www.r-project.org).

## Measuring Risk and Managing Uncertainty

Quantifying the uncertainty in prediction has been a neglected area in the field of malaria mapping and in ecology, more generally [49]. Our aim is to present all risk maps generated through MAP with uncertainty guides, companion maps that show the spatial variation in predictive accuracy. We also intend to evaluate the most-accurate procedures for achieving the basic mapping with appropriate robustness measures [11,50–54]. There are alternative methods available to achieve mapping with error estimations: Bayesian [11,21,55], discriminant analyses [52], and logistic-regression [50,52] techniques, among others [53,54], and these will all be systematically tested as part of the MAP project. The code written to implement these techniques will again be written in R and distributed freely upon acceptance of its products through peer review.

## Where People Live

Accurate population data are critical for the assessment of the effects of human population density on malaria risk and the attribution of risk to populations [56]. These databases are also becoming increasingly accessible [47,56,57]. Areas of the world for which we have a particularly poor understanding of human population distribution will limit the accuracy of MAP and other databases to derive population at-risk estimates. Countries of specific concern are highlighted in the ancillary data section of the MAP Web site. More contemporary or higher spatial resolution census data supplied to MAP will be forwarded with permission to collaborators developing the Gridded Population of the World, version 3 (GPWv3) and the Global Rural–Urban Mapping Project (GRUMP) (http://sedac.ciesin.columbia.edu).

## Future Applications

These planned malaria-endemicity maps will provide the basis for increasing the fidelity of morbidity [4], mortality [5,6] and co-infection burden estimates [58,59]. These studies lead logically to more-accurate commodity demand and budget estimation. As we have argued, these maps may also provide a means to help determine the distribution of intervention types and mixes within countries. Companion maps of the global distribution of the main anopheline vector species will also be particularly important in helping inform the appropriate modes of control. At the very least, these map suites should augment the objective monitoring and evaluation of our interventions in the coming years. There are strong arguments for these exercises being conducted independently of international agencies responsible for the implementation and evaluation of interventions [60–62].

While we strive to assemble data to define an endemicity baseline, the static maps we generate will represent a “snapshot” of a dynamic malaria epidemiology. It is important to establish this epidemiological baseline because history has shown that changes independent of planned interventions are inevitable [63]. These global environmental changes will affect the populations at risk of malaria. Land-use changes [64], such as deforestation [65], may modify vector population dynamics, for example. Population growth, urbanization [6], and climate change [66] will additionally affect human population dynamics. Other factors such as the progression of the HIV/AIDS pandemic and changes in undernutrition and socioeconomic status [66,67] will influence the ability of human population to cope with malaria infection. These changes will be significant over the time span of international development goals and targets. MAP aims to develop plausible scenarios for many of these influences and techniques to model their potential impact, as they will be confounders in our ability to evaluate critically interventions at scale.

## Conclusions

The distribution of populations exposed to the risk of P. falciparum and P. vivax malaria is poorly understood at the global level. Considerable effort as part of MAP is required to improve our basic maps of malaria transmission intensity and help identify the global distribution of populations at risk of malaria. This will involve assembling the largest-ever collection of PR data and a significant parallel investment in establishing the required environmental, population, and malaria vector data.

A very considerable research effort is also required to evaluate those statistical techniques needed to relate the PR and environmental data for extensive map predictions with confidence intervals. This will take time and succeed only with the cooperation of the malaria control community. To encourage interaction, MAP will be connected with the philosophy of open access, so that all data collected and techniques developed can be made available in the public domain rapidly after peer review.

## Abbreviations

MAP: Malaria Atlas Project
PR: parasite rate
